# Clinical and economic benefits of seasonal COVID-19 vaccination in Germany: results from the ROUTINE-COV19 Study, September 2022 to March 2024

**DOI:** 10.2807/1560-7917.ES.2026.31.15.2500672

**Published:** 2026-04-16

**Authors:** Andrea Schmetz, Julia Knaul, Sabrina Müller, Thomas Wilke, Jingyan Yang, Christoph Spinner, Clara Lehmann

**Affiliations:** 1 BioNTech SE, Mainz, Germany; 2 GIPAM GmbH, Wismar, Germany; 3 The Institute for Pharmacoeconomics and Drug Logistics (IPAM), University of Wismar, Wismar, Germany; 4 Pfizer Inc., New York, United States; 5 Technical University of Munich (TUM) School of Medicine and Health – Clinical Department of Internal Medicine II, TUM University Hospital, Munich, Germany; 6 Department I of Internal Medicine, Medical Faculty and University Hospital Cologne, University of Cologne, Cologne, Germany; 7 Center for Molecular Medicine Cologne (CMMC), Medical Faculty and University Hospital Cologne, University of Cologne, Cologne, Germany; 8 German Center for Infection Research (DZIF), Bonn-Cologne, Germany

**Keywords:** COVID-19 endemic phase, COVID-19 case severity, in-hospital COVID-19 case mortality, healthcare burden, economic impact, statutory health insurance data, Germany, seasonal vaccination

## Abstract

**BACKGROUND:**

Vaccinations against COVID-19 were integrated into routine care in Germany in April 2023. However, evidence of the impact of seasonal vaccination remains limited.

**AIM:**

To assess the clinical and economic impact of COVID-19 vaccination in routine care during the early SARS-CoV-2-endemic phase in Germany.

**METHODS:**

A retrospective cohort study using statutory health insurance data from two German federal states (Saxony and Thuringia), covering over 3 million individuals, was conducted. Adults aged ≥ 18 years vaccinated against COVID-19 between 1 September and 30 November 2023 were matched 1:1 with unvaccinated individuals using propensity scores. Outcomes during the 4-month follow-up included occurrence of SARS-CoV-2 infection, long COVID, other respiratory infections, hospitalisations, mortality, healthcare costs and indirect costs caused by sick leave. Rate and hazard ratios (RR, HR) with 95% confidence intervals (CI) were calculated. Sensitivity analyses tested robustness.

**RESULTS:**

A total of 146,132 individuals (73,066 per group) were matched. COVID-19 vaccination was associated with reduced rates of long COVID (RR: 0.43; 95% CI: 0.26–0.70), respiratory infections (RR: 0.91; 95% CI: 0.87–0.95) and COVID-19-related hospitalisations (RR: 0.41; 95% CI: 0.31–0.54). All-cause mortality was 25% lower among COVID-19-vaccinated individuals (HR: 0.76; 95% CI: 0.70–0.82). Healthcare costs were lower in the vaccinated cohort, particularly for inpatient care, e.g. EUR 1 million savings in COVID-19-related hospitalisations. Indirect costs caused by sick leave were also reduced by EUR 1.3 million.

**CONCLUSION:**

Seasonal COVID-19 vaccinations in routine care settings were associated with substantial clinical and economic benefits. These real-world findings support continued implementation of national immunisation recommendations during the endemic phase of SARS-CoV-2 circulation.

**Key public health message**

**What did you want to address in this study and why?**

In Germany, COVID-19 vaccinations were integrated into routine care and thereby included in the statutory health insurance records in April 2023, allowing real-world clinical and economic assessment. We wanted to analyse the impact of autumn COVID-19 vaccinations following integration into routine care, using data from adults aged ≥ 18 years insured in the federal states of Saxony and Thuringia between September 2022 and March 2024.

**What have we learnt from this study?**

COVID-19-vaccinated individuals experienced lower hospitalisation rates associated with COVID-19, respiratory or cardiovascular diseases, as well as reduced all-cause mortality and long COVID diagnoses. This translated to lower healthcare costs and fewer sick leave days compared with unvaccinated individuals, with estimated savings of ca EUR 1 million in inpatient care and EUR 1.3 million in indirect costs caused by sick leave in the 4-month follow-up period.

**What are the implications of your findings for public health?**

With SARS-CoV-2 remaining endemic, our findings highlight the continued health and economic benefits of COVID-19 vaccination. These results support the sustained implementation of national vaccination recommendations and provide evidence to guide policy decisions in Germany and other countries with similar healthcare systems.

## Introduction

While COVID-19 is no longer considered a global public health emergency [1], the severe acute respiratory syndrome coronavirus 2 (SARS-CoV-2) continues to circulate globally as an endemic pathogen, evolving through mutations and causing both symptomatic and severe infections. Vulnerable populations, including older adults, immunocompromised individuals, and those with relevant chronic comorbidities, are disproportionately affected [2]. For example, in 2022, ca 200,000 COVID-19-associated and ca 55,000 influenza-associated severe acute respiratory infection (SARI) hospitalisations were documented in Germany. In subsequent years, the number of COVID-19-associated hospitalisations has remained of a similar magnitude to those observed for influenza [3].

COVID-19 vaccines continue to be an effective tool to prevent serious illness, hospitalisation and death from COVID-19 [4]. To mitigate the impact of disease, countries have adapted their prevention and immunisation strategies over time. In 2023, the German Standing Committee on Vaccination (Staendige Impfkommission; STIKO) at the German national public health insitute, the Robert Koch Institute (RKI), updated its COVID-19 vaccination guidelines to include baseline immunisation for all individuals aged 18 years and older, as well as annual, variant-adapted vaccines in autumn for individuals aged 60 years and older, those with high-risk conditions, residents of long-term care facilities, immunocompromised individuals and their close contacts and healthcare personnel [5,6]. Despite these recommendations, COVID-19 vaccination rates in Germany remain low, with only 20.9% of individuals aged 60 or older and 14.3% of individuals with comorbidities vaccinated during the 2024/25 autumn and winter seasons [7]. Similar trends of limited vaccination uptake during the 2023/24 season were observed across several European Union/European Economic Area (EU/EEA) countries, as reported by the European Centre for Disease Prevention and Control (ECDC) [8].

Since April 2023, COVID-19 vaccination has been integrated into routine care in Germany, enabling structured documentation in statutory health insurance (SHI) data and facilitating real-world evaluation of vaccination impact. For the 2023/24 season, two variant-adapted mRNA vaccines, Spikevax (mRNA-1273, Moderna) XBB.1.5 and Comirnaty (BNT162b2, BioNTech-Pfizer) XBB.1.5, were authorised in the European Union [9,10]. Both were recommended by STIKO in Germany; however, Comirnaty was the most widely used vaccine nationwide in 2023 and 2024 (> 90% of vaccinations) [6,11]. While clinical trials have demonstrated their safety and efficacy [12,13], little is known about their real-world impact in Germany after integration into routine standard care.

The ROUTINE-COV19 study is a large retrospective cohort study based on SHI data from more than 3 million insured individuals in Saxony and Thuringia, two federal states in eastern Germany. Using data from ROUTINE-COV19, the present analysis examines clinical and economic outcomes in vaccinated vs unvaccinated individuals following the integration of routine COVID-19 vaccination into care in Germany. These analyses build on previous results describing the epidemiology and healthcare utilisation of COVID-19 in the early endemic phase, highlighting seasonal peaks and substantial healthcare utilisation, particularly among high-risk groups [14]. 

## Methods

### Study design and analysis periods

This comparative analysis is part of the ROUTINE-COV19 study, a retrospective, real-world study based on German claims data provided by a regional SHI fund (AOK PLUS), which covers more than 3.4 million individuals in two German states, accounting for ca 50% of the population in these states and about 5% of the total German SHI population.

**Figure 1:**
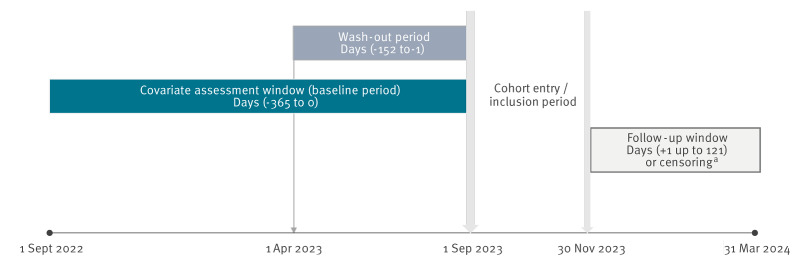
Analysis periods of the ROUTINE-COV19 study, Saxony and Thuringia, Germany, 1 September 2022–31 March 2024

^a^ Censoring is applied at the earliest occurrence of one of the following events: death (if not the outcome of interest in a given analysis), loss to follow-up (end of insurance), or the end of the study period (31 March 2024).

For this analysis, anonymised data covering the entire ensured population of the cooperating SHI from 1 September 2022 to 31 March 2024 were available (Figure 1). The dataset contained comprehensive information on outpatient and inpatient services, pharmaceutical treatments, diagnostic codes, procedures, vaccination records, rehabilitation care, work absences and demographic characteristics, including age and sex. Comorbidities used for adjustment in the comparative analyses were derived from outpatient and inpatient diagnoses based on International Statistical Classification of Diseases and Related Health Problems, 10^th^ Revision, German Modification (ICD-10-GM) codes [15].

### Study population

The study population was initially defined as all individuals insured by AOK PLUS on 30 November 2023. From this population, individuals were included if they had continuous insurance coverage during the inclusion period (1 Sep–30 Nov 2023) and the preceding 12-month baseline period (from 1 Sep 2022).

In this study, ‘vaccinated’ refers to individuals vaccinated against SARS-CoV-2 during the inclusion period, regardless of vaccine product, unless otherwise specified. The vaccination cohort comprised all individuals who had a vaccination record identified via dedicated outpatient billing codes (EBM) in the claims data during the inclusion period from 1 September to 30 November 2023. This period was selected to allow sufficient time after the introduction of the EBM code for COVID-19 vaccination in April 2023, ensuring complete capture within routine care. It also corresponds to the general vaccination season in Germany, when seasonally adapted vaccines were available. December was not included in the inclusion period, as only a few additional vaccinations were recorded, and the dataset was restricted to March 2024. Consequently, December was assigned to the follow-up period to provide a 4-month follow-up (1 December 2023–31 March 2024), with censoring in the event of death or termination of insurance coverage.

Individuals were excluded if they lacked continuous insurance coverage during the inclusion period (1 September–30 November 2023) or during the 12-month baseline period preceding the inclusion period, which was used for covariate assessment (Figure 1). Equally, individuals with an inpatient or outpatient diagnosis of long COVID (ICD-10-GM: U09.9!) during the baseline or inclusion period were excluded to avoid potential bias related to pre-existing post-COVID-19 conditions. Long COVID represents a post-acute sequela of previous infection that may lead to chronically increased healthcare utilisation and could confound the assessment of outcomes. Individuals with a COVID-19 vaccination record during the wash-out period (1 April–31 August 2023) were excluded to ensure that all vaccinated individuals in the study received a vaccination after the introduction of the seasonally adapted XBB.1.5 vaccines. Vaccinations administered during the wash-out period will have included earlier vaccine formulations and were therefore excluded to maintain a homogeneous vaccinated cohort.

### Sensitivity analyses 

Using these common exclusion criteria, the base case sample was defined. The base case analysis applied uniform, less restrictive exclusion criteria to avoid bias from asymmetric exclusions between cohorts. More strict exclusion criteria were tested in sensitivity analyses: (i) Sensitivity Scenario A: in addition to exclusion criteria applied in the base case sample selection, individuals with an ICD-10-GM code U11.9 (‘Need for immunisation against COVID-19, unspecified’) were excluded, as this code was frequently used as a proxy for COVID-19 vaccination before the introduction of dedicated EBM billing codes; (ii) Sensitivity Scenario B: all base case selection criteria, but individuals with a COVID-19 diagnosis during the washout period were additionally excluded; for unvaccinated individuals, this exclusion extended to the inclusion period; (iii) Sensitivity Scenario C: all base case selection criteria were applied and supplemented by the exclusion of individuals who received a COVID-19 vaccination during the follow-up period; (iv) Sensitivity Scenario D: exclusion of individuals vaccinated in the follow-up period and restriction of the inclusion period to October 2023.

The deviations from the base case applied in each sensitivity scenario are summarised in Supplementary Table S1.

### Population-adjustment method for comparing vaccinated and unvaccinated cohorts

To reduce confounding from the non-random allocation of COVID-19 vaccination, propensity score matching (PSM) was applied to create comparable cohorts of vaccinated and unvaccinated individuals. Propensity scores were estimated using a logistic regression model that included key baseline covariates: age, sex, comorbidity profile (Charlson Comorbidity Index, CCI [16]) and receipt of influenza vaccination.

Matching was performed using a nearest-neighbour algorithm without replacement and a caliper of 0.0001. Multiple scenarios were tested and evaluated to ensure the best possible matching of vaccinated and unvaccinated cohorts. In general, a 1:1 matching ratio was applied. Additionally, for the base case sample and Sensitivity Scenario C, a 1:3 matching ratio was performed as a supplementary approach to assess the impact of sample size on precision and effect estimates.

Matching quality was evaluated using standardised mean differences (SMDs), with SMD values below 10% considered acceptable. Additionally, Rubin’s B statistic (the absolute standardised difference in linear propensity score means) was assessed, with values below 25 indicating adequate covariate balance.

### Outcome assessment

Patient characteristics were assessed based on claims data from the 12-month baseline period preceding cohort entry (see Figure 1). Available information from the SHI claims dataset included demographics and pre-existing medical conditions. Demographic variables comprised age, sex and employment status. The extent of comorbidity was evaluated using both the CCI [16] and the Elixhauser Comorbidity Index [17]. Cardiovascular risk was further characterised using the CHA_2_DS_2_-VASc score [18] and the documented presence of specific cardiovascular diagnoses, i.e. atrial fibrillation, heart failure and coronary heart disease. In addition, the prevalence of depression and anxiety disorders was described and compared between groups. 

The outcomes of interest compared between the matched vaccinated and unvaccinated cohorts during the follow-up period were: (i) documented SARS-CoV-2 infection, identified via ICD-10-GM codes U07.1! or U07.2! (for the definition of new COVID-19 cases, please refer to the previously published ROUTINE-COV19 study results [14]); (ii) incident diagnoses of long COVID, identified by confirmed outpatient or inpatient diagnosis using ICD-10-GM code U09.9!; (iii) respiratory infections, identified by confirmed outpatient or inpatient diagnosis using ICD-10-GM codes A15, A16, J00-J06, J09-J18, J20-J22, J40, J44.0, and J44.1. Respiratory infections were analysed separately from SARS-CoV-2 infections to differentiate the direct protective effect of vaccination against COVID-19 from potential broader effects on respiratory morbidity. As routine SARS-CoV-2 testing was no longer systematically performed during the study period, some undetected COVID-19–related cases may have been included in this category; (iv) COVID-19-related hospitalisations, defined as hospital admissions with a confirmed COVID-19 diagnosis (ICD-10-GM: U07.1!) and either a predefined main diagnosis—such as pneumonia, chronic lower respiratory disease, other respiratory infections, heart failure, chronic heart disease, acute pericarditis/myocarditis, or atrial fibrillation—or a documented requirement for mechanical ventilation, irrespective of the main diagnosis [14]; (v) respiratory- and cardiovascular disease (CVD)-related hospitalisations; (vi) all-cause mortality; (vii) mortality during COVID-19-related hospitalisation; (viii) COVID-19-related healthcare resource utilisation (HCRU) and associated costs, including general practitioner (GP) visits, outpatient specialist visits, and hospitalisations/days related to a COVID-19 diagnosis; (ix) costs of respiratory- and cardiovascular-related hospitalisations; (x) indirect costs calculated based on the number of sick leave days due to COVID-19 or respiratory infections among working-age individuals (18–66 years), using official average productivity loss estimates provided by the Federal Institute for Occupational Safety and Health (BAuA [19]).

### Statistical analysis

Descriptive statistics were used to summarise characteristics of vaccinated and unvaccinated cohorts. Continuous variables were reported as means, standard deviations (SD) and medians; group comparisons were conducted using Student’s t-tests. Categorical variables were presented as frequencies and compared using the Chi-square test or, where appropriate, Fisher’s exact test.

Effect estimates were calculated as rate ratios (RRs) or hazard ratios (HRs), depending on the outcome type. Event rates were expressed as events per 100 person-years. For time-to-event outcomes, Cox proportional hazards models were applied to estimate HRs with 95% confidence intervals (CIs).

All statistical tests were two-sided, and a p value < 0.05 was considered statistically significant. Analyses were performed using STATA/MP version 14 (StataCorp LLC) and Microsoft SQL Server 2014.

## Results

In the base case sample selection, a total of 73,067 individuals vaccinated against COVID-19 and 3,145,952 unvaccinated individuals met the eligibility criteria before matching. Individuals vaccinated during the wash-out period were excluded from the analysis sample used for later matching. These excluded individuals (n = 2,069) were older on average (mean age: 70.3 years) and had higher comorbidity scores than the study cohorts (Table 1), consistent with the prioritisation of older (≥ 60 years) and high-risk individuals for early vaccination.

**Table 1:** Baseline characteristics of vaccinated and unvaccinated cohorts, Saxony and Thuringia, Germany, September 2022–September 2023 (matched analysis sample: n = 73,066 per group)

Characteristics	Individuals excluded in the wash-out period (not considered for matched comparison)		Unmatched					Matched				
			Vaccinated cohort		Unvaccinated cohort		SMD x 100 (% bias)	Vaccinated cohort		Unvaccinated cohort		SMD x 100 (% bias)
	n = 2,069		n = 73,067		n = 3,145,952			n = 73,066		n = 73,066		
	n	%	n	%	n	%		n	%	n	%	
Age in years: mean (SD); median	70.3 (17.3); 73		71.9 (13.6); 73		44.4 (24.5); 45		138.7	71.9 (13.6); 73		71.9 (13.5); 73		0.0
Female sex	1,051	50.8	36,647	50.2	1,649,594	52.4	−4.6	36,647	50.2	36,656	50.2	0.0
Male sex	1,018	49.2	36,420	49.8	1,496,358	47.6	4.6	36,419	49.8	36,410	49.8	0.0
Employment status/’type of insurance’												
Employee/self-payer	369	17.8	12,898	17.7	1,491,441	47.4	−67	12,898	17.7	14,218	19.5	−3.7
Pensioner/retiree or rehabilitator^a^	1,561	75.4	57,239	78.3	806,017	25.6	124.4	57,238	78.3	56,159	76.9	3.4
Unemployed	82	4	2,030	2.8	184,268	5.9	−15.2	2,030	2.8	1,860	2.5	0.7
Insured family member without own income	57	2.8	900	1.2	664,226	21.1	−66.5	900	1.2	829	1.1	0.2
Comorbidity scores: mean (SD); median												
Charlson comorbidity index (CCI)	3.2 (2.9); 3		2.9 (2.7); 2		1.0 (1.9); 0		84.0	2.9 (2.7); 2		2.9 (2.7); 2		0.1
Elixhauser comorbidity index	8.2 (9.4); 6		7.5 (9.1); 5		2.3 (6.2); 0		66.4	7.5 (9.1); 5		7.5 (9.1); 5		0.2
CHA2DS2-VASc (cardiovascular risk score)	1.8 (1.3); 2		1.7 (1.2); 2		0.6 (1.0); 0		93.8	1.7 (1.2); 2		1.7 (1.2); 2		−1.9
Baseline clinical characteristics												
Presence of AF	341	16.5	11,017	15.1	128,512	4.1	38.0	11,017	15.1	10,789	14.8	0.8
Presence of HF	411	19.9	13,048	17.9	167,497	5.3	39.9	13,047	17.9	13,797	18.9	−3.4
Presence of CHD	468	22.6	16,011	21.9	206,239	6.6	45.1	16,010	21.9	16,563	22.7	−2.6
Presence of depression	413	20	12,019	16.4	290,284	9.2	21.7	12,019	16.4	10,693	14.6	4.9
Presence of anxiety disorder	227	11	6,588	9	199,897	6.4	10.0	6,588	9.0	6,374	8.7	1.3
Influenza vaccinated	1,392	67.3	46,678	63.9	498,989	15.9	112.5	46,677	63.9	46,667	63.9	0.0
COVID-19 diagnosis in the baseline period	155	7.5	4,348	6.00	188,761	6.0	5.6	4,348	6.0	4,087	5.6	3.4

AF: atrial fibrillation; CHA_2_DS_2_-VASc score: Congestive heart failure, Hypertension, Age ≥75 years, Diabetes mellitus, Stroke/transient ischaemic attack - Vascular disease, Age 65–74 years, Sex category cardiovascular risk score; CHD: coronary heart disease; HF: heart failure; N/R: not reported; SD: standard deviation; SMD: standardised mean difference.

^a^ Individuals receiving rehabilitation benefits under statutory health/pension insurance.

All variables were statistically significantly different (p < 0.05) between the vaccinated and unvaccinated cohorts before matching, with the exception of ‘Employment status: Rehabilitator.’ After matching, none of the characteristics were significantly different between the cohorts. Balance between cohorts before and after matching was assessed using standardised mean differences (SMDs), as well as t-tests (continuous variables) and Chi-square tests (categorical variables).

After applying 1:1 PSM, 73,066 of 73,067 vaccinated individuals were successfully matched to an unvaccinated individual and retained in each cohort, contributing a total of 24,015 person-years in the vaccinated group and 23,943 person-years in the unvaccinated group. The quality of the matching process was confirmed through key balancing diagnostics (mean/maximum standardised mean difference and Rubin’s B) based on all baseline characteristics shown in Table 1; the maximum standardised mean difference across characteristics was reduced to < 5%, the mean standardised difference was 1.7 and Rubin’s B was 10.3, indicating excellent covariate balance between the matched cohorts (see also Supplementary Figure S1). One individual was excluded due to matching constraints.

Prior to matching, substantial differences were observed between the vaccinated and unvaccinated cohorts (Table 1). Vaccinated individuals were markedly older, with a mean age of 71.9 years compared with 44.4 years in the unvaccinated group. The vaccinated cohort also had significantly higher comorbidity scores, with a mean CCI of 2.9 versus 1.0, and a mean Elixhauser Comorbidity Index of 7.5 vs 2.3. Cardiovascular risk was more pronounced in the vaccinated cohort, as reflected by a mean CHA_2_DS_2_-VASc score of 1.7 vs 0.6, and a higher prevalence of atrial fibrillation (15.1 vs 4.1%), heart failure (17.9 vs 5.3%), and coronary heart disease (21.9 vs 6.6%). In addition, vaccinated individuals were more likely to have documented depression (16.4 vs 9.2%) and anxiety disorders (9.0 vs 6.4%). Influenza vaccination was also more common among vaccinated individuals (63.9 vs 15.9%), further suggesting differences in the risk profile and healthcare-seeking behaviour between the groups.

After matching, the vaccinated and unvaccinated cohorts were highly comparable across all assessed characteristics. In the matched population (n = 73,066 per group), the mean age in both groups was 71.9 years, with 50.2% female individuals in each cohort. The mean CCI and Elixhauser indices were identical between groups (CCI: 2.9, Elixhauser: 7.5), as were the mean CHA_2_DS_2_-VASc scores (1.7). Prevalence of atrial fibrillation (15.1 vs 14.8%), heart failure (17.9 vs 18.9%), coronary heart disease (21.9 vs 22.7%), depression (16.4 vs 14.6%), and anxiety (9.0 vs 8.7%) closely aligned. The proportion of individuals vaccinated against influenza was identical post-matching (63.9% in both cohorts).

### Comparison of event rates in vaccinated and unvaccinated cohorts

**Figure 2:**
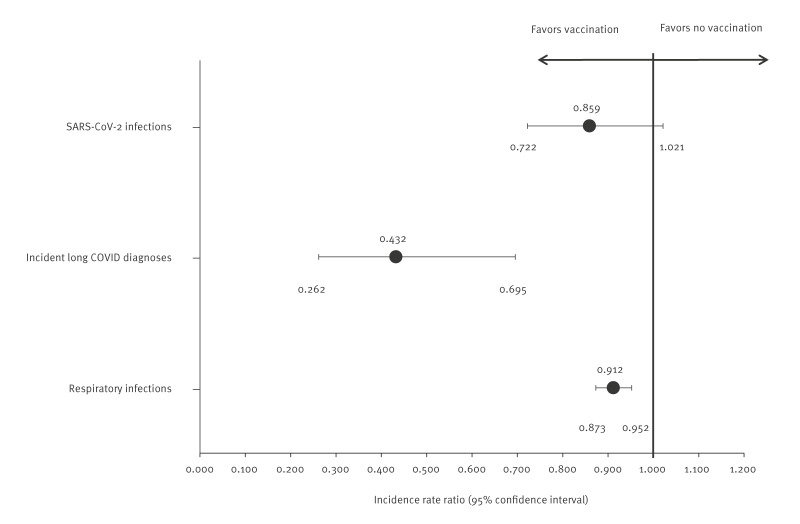
Forest plot of rate ratios comparing SARS-CoV-2 infections, incident long COVID diagnoses and respiratory infections of vaccinated and unvaccinated cohorts, Saxony and Thuringia, Germany, 1 December 2023–31 March 2024 (n = 73,066 per group)

In the matched vaccinated and unvaccinated groups, differences were observed across key clinical outcomes. In the base case scenario, the rate of documented SARS-CoV-2 infections was 1.04 per 100 patient-years in the vaccinated group vs 1.21 in the unvaccinated group, corresponding to an RR of 0.86 (95% CI: 0.72–1.02, p = 0.079; Figure 2). For incident long COVID diagnoses, event rates were 0.11 vs 0.25 per 100 patient-years, yielding an RR of 0.43 (95% CI: 0.26–0.70, p < 0.001). Respiratory infections occurred at a rate of 16.61 in the vaccinated cohort compared with 18.22 in the unvaccinated cohort, with an RR of 0.91 (95% CI: 0.87–0.95, p < 0.001).

SARS-CoV-2: severe acute respiratory syndrome coronavirus 2.

Rate ratios are based on observed events per person-time during the 4-month follow-up period (1 Dec 2023–31 Mar 2024). Estimates compare matched vaccinated and unvaccinated cohorts (n = 73,066 per group). Error bars represent 95% confidence intervals.

Comparable rate ratios and patterns were observed across all sensitivity scenarios, which are provided in Supplementary Figure S2.

### Severe outcomes and hospital stays in vaccinated and unvaccinated cohorts

**Figure 3:**
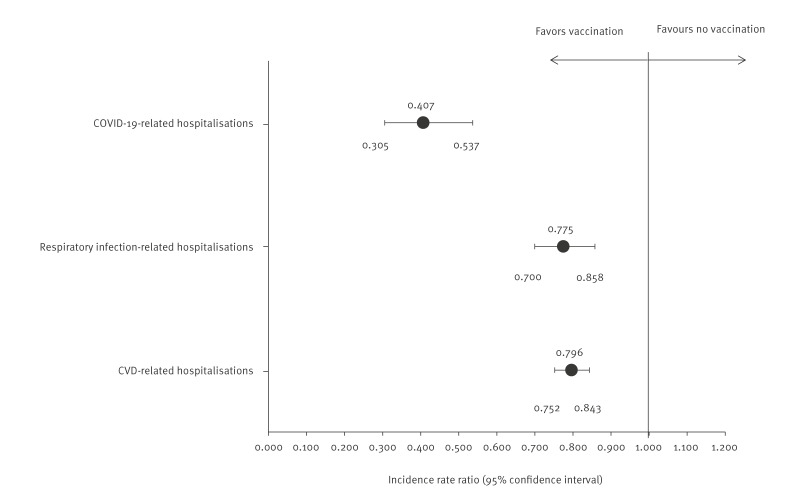
Forest plot of rate ratios comparing COVID-19-related, respiratory infection-related and CVD-related hospitalisations of vaccinated and unvaccinated cohorts, Saxony and Thuringia, Germany, 1 December 2023–31 March 2024 (n = 73,066 per group)

We analysed COVID-19-related, respiratory infection-related and CVD-related hospitalisations during the follow-up period in both cohorts. In the follow-up period, event rates were consistently lower among vaccinated individuals in the base case scenario (Figure 3) and across all other scenarios, which are presented in Supplementary Figure S3. The rate of COVID-19-related hospitalisations was 0.30 per 100 patient-years in the vaccinated cohort compared with 0.75 in the unvaccinated group, corresponding to an RR of 0.41 (95% CI: 0.31–0.54, p < 0.001). COVID-19-related hospitalisation rates were primarily driven by inpatient stays with a main diagnosis of pneumonia, occurring at a rate of 0.14 per 100 patient-years in the vaccinated cohort compared with 0.34 per 100 patient-years in the unvaccinated cohort, corresponding to an RR of 0.41 (95% CI: 0.27–0.62, p < 0.001). For respiratory infection-related hospitalisations, event rates were 2.80 vs 3.62 per 100 person-years, yielding an RR of 0.778 (95% CI: 0.70–0.86, p < 0.001). The rate of CVD-related hospitalisations was 8.89 in the vaccinated cohort and 11.17 in the unvaccinated group, with a corresponding RR of 0.80 (95% CI: 0.75–0.84, p < 0.001).

CVD: cardiovascular disease.

Rate ratios are based on observed events per person-time during the 4-month follow-up period (1 Dec 2023–31 Mar 2024). Estimates compare matched vaccinated and unvaccinated cohorts (n = 73,066 per group). Error bars represent 95% confidence intervals.

### All-cause and in-hospital mortality in vaccinated and unvaccinated cohorts

**Figure 4:**
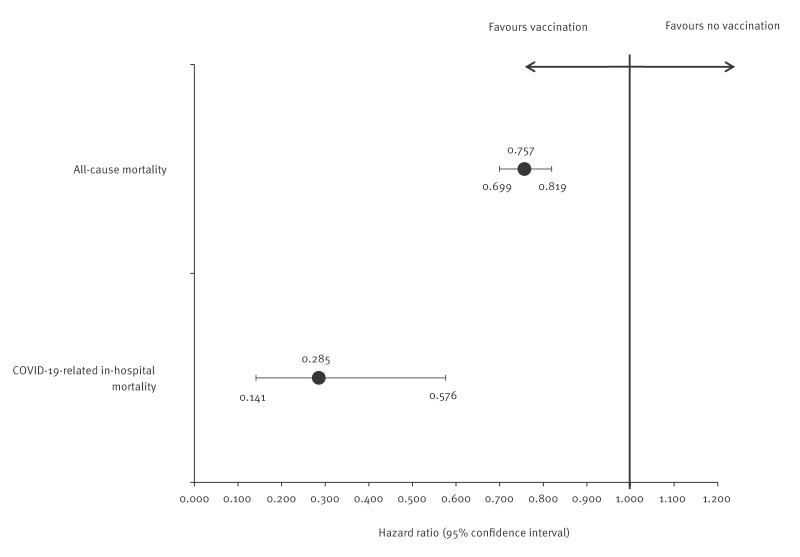
Forest plot of hazard ratios comparing all-cause and COVID-19-related in-hospital mortality of vaccinated and unvaccinated cohorts, Saxony and Thuringia, Germany, 1 December 2023–31 March 2024 (n = 73,066 per group)

During the 4-month follow-up, all-cause mortality was substantially lower among vaccinated individuals, with an event rate of 4.52 per 100 patient-years compared with 6.01 per 100 patient-years in the unvaccinated cohort. This difference corresponded to an HR for time to all-cause death of 0.756 (95% CI: 0.70–0.82, p < 0.001) (Figure 4). The reduction in all-cause mortality remained statistically significant and consistent across all sensitivity analyses, which are provided in Supplementary Figure S4.

Hazard ratios are based on time-to-event analyses during the 4-month follow-up period (1 Dec 2023–31 Mar 2024). Error bars represent 95% confidence intervals.

Mortality during COVID-19-related hospitalisations also appeared lower in the vaccinated cohort. The rate of death during COVID-19-related hospitalisation was 0.07 per 100 patient-years among vaccinated individuals, compared with 0.27 per 100 patient-years in the unvaccinated group, translating to an HR of 0.29 (95% CI: 0.14–0.58, p < 0.001). Of note, the results for COVID-19-related in-hospital mortality were less stable across sensitivity scenarios, with wider confidence intervals and non-significant results in Sensitivity Scenarios B and D (see Supplementary Figure S4 for all sensitivity scenarios).

### Comparison of healthcare resource utilisation and associated costs in vaccinated and unvaccinated cohorts

Vaccinated individuals used COVID-19-related outpatient care slightly more frequently: 0.23 GP visits per person-year vs 0.207 in the unvaccinated group, with associated per-person-year costs of EUR 4.87 vs EUR 4.75, respectively (Table 2). Specialist visits were more frequent among the vaccinated group (0.18 vs 0.10 per person-year), corresponding to EUR 4.51 in the unvaccinated group vs EUR 2.72 in the vaccinated group in specialist care costs.

**Table 2:** COVID-19-related healthcare resource utilisation and associated costs during the follow-up period, Saxony and Thuringia, Germany 1 December 2023–31 March 2024 (n = 73,066 per group)

Characteristics	Total amounts incurred in the follow-up period			Amounts per observed person-years		
	Vaccinated	Unvaccinated	Difference: vaccinated − unvaccinated	Vaccinated	Unvaccinated	Difference: vaccinated − unvaccinated
COVID-19-related outpatient GP visits						
Number of visits	5,402	4,945	457	0.225 ppy	0.207 ppy	0.018 ppy
Cost	EUR 116,974.62	EUR 113,788.12	EUR 3,186.50	EUR 4.87 ppy	EUR 4.75 ppy	EUR 0.12 ppy
COVID-19-related outpatient specialist visits						
Number of visits	4,268	2,427	1,841	0.178 ppy	0.101 ppy	0.076 ppy
Cost	EUR 108,371.04	EUR 65,009.89	EUR 43,361.15	EUR 4.51 ppy	EUR 2.72 ppy	EUR 1.80 ppy
COVID-19-related hospitalisations						
Number of admissions	73	179	−106	0.003 ppy	0.007 ppy	−0.004 ppy
Hospitalisation days	617	1,729	−1,112	0.026 ppy	0.072 ppy	−0.047 ppy
Cost	EUR 507,394.43	EUR 1,542,585.82	EUR −1,035,191.39	EUR 21.13 ppy	EUR 64.43 ppy	EUR −43.30 ppy
Respiratory infection-related hospitalisations						
Number of admissions	673	866	−193	0.028 ppy	0.036 ppy	−0.008 ppy
Hospitalisation days	5,065	6,781	−1,716	0.211 ppy	0.283 ppy	−0.072 ppy
Cost	EUR 3,894,497.77	EUR 5,642,975.82	EUR −1,748,478.05	EUR 162.17 ppy	EUR 235.69 ppy	EUR −73.52 ppy
CVD-related hospitalisations						
Number of admissions	2,136	2,674	−538	0.089 ppy	0.112 ppy	−0.023 ppy
Hospitalisation days	13,276	17,595	−4,319	0.553 ppy	0.735 ppy	−0.182 ppy
Cost	EUR 13,926,673.02	EUR 19,173,242.30	EUR −5,246,569.28	EUR 579.91 ppy	EUR 800.80 ppy	EUR −220.89 ppy
Sick leave days in individuals of working age						
Number of working-age individuals	21,729	21,719	+10	7,172 ppy	7,171 ppy	+1 ppy
Number of sick leave days caused by COVID-19	3,954	5,819	−1,865	0.551 ppy	0.811 ppy	−0.260 ppy
Indirect costs of sick leave days caused by COVID-19	EUR 525,882.00	EUR 773,927.00	EUR −248,045.00	EUR 73.33 ppy	EUR 107.92 ppy	EUR −34.60 ppy
Number of sick leave days caused by respiratory infections	31,818	39,596	−7,778	4.437 ppy	5.522 ppy	−1.085 ppy
Indirect costs of sick leave days caused by respiratory infections	EUR 4,231,794.00	EUR 5,266,268.00	EUR −1,034,474.00	EUR 590.06 ppy	EUR 734.38 ppy	EUR −44.32 ppy

CVD: cardiovascular disease; GP: general practitioner; EUR: euro; ppy: per person-year.

COVID-19-related inpatient care, by contrast, was more frequently used by unvaccinated individuals. Vaccinated individuals experienced fewer COVID-19-related hospitalisation days (617 vs 1,729 days), resulting in substantially lower inpatient costs (Table 2). The per-person-year costs of COVID-19-related hospitalisations were EUR 21.13 in the vaccinated cohort compared with EUR 64.43 in the unvaccinated group, corresponding to a cost saving of EUR 43.30 per person-year. Over the 4-month follow-up period, this translated into a total cost reduction of EUR 1,035,191.39 in COVID-19-related inpatient care costs in favour of vaccinated individuals.

The number of hospitalisations caused by respiratory infections in general was also notably lower among vaccinated individuals. Over the follow-up period, 5,065 hospitalisation days were observed in the vaccinated cohort, compared with 6,781 days in the unvaccinated cohort. The mean per-person-year cost for respiratory infection-related hospitalisations was EUR 162.17 in the vaccinated group vs EUR 235.69 in the unvaccinated group, resulting in a difference of EUR 73.52 per person-year.

Similarly, CVD-related hospitalisations were less common in the vaccinated group (2,136 vs 2,674 events) and were associated with a lower cumulative number of hospital days (13,276 vs 17,595 days). On a per-person-year basis, CVD-related hospitalisation costs were EUR 579.91 in the vaccinated cohort and EUR 800.80 in the unvaccinated cohort, yielding a cost reduction of EUR 220.89 per person-year.

### Impact of seasonal COVID-19 vaccination on sick leave 

The vaccinated and unvaccinated cohorts comprised 21,729 and 21,719 individuals of working age (18–66 years), respectively. Work absenteeism and related indirect costs were evaluated within these cohorts. The total follow-up time was 7,172 and 7,171 person-years, respectively.

Across the 4-month follow-up period, vaccinated individuals exhibited fewer days of sick leave for COVID-19 (3,954 vs 5,819 days) and respiratory infections (31,818 vs 39,596 days) than their unvaccinated counterparts (Table 2). When adjusted for person-time, the number of sick leave days per person-year was 0.55 in vaccinated individuals vs 0.81 in unvaccinated individuals for COVID-19 and 4.44 vs 5.52 for respiratory infections, indicating absolute reductions of 0.26 and 1.09 days per person-year, respectively. These reductions translated into lower indirect costs due to sick leave for the vaccinated cohort.

Indirect costs caused by COVID-19-related sick leave amounted to EUR 73.33 per person-year among vaccinated individuals compared with EUR 107.92 in the unvaccinated group, representing a difference of EUR 34.60 per person-year and a total reduction of EUR 248,045.00 over the 4-month follow-up period. For respiratory infection-related absenteeism, estimated indirect costs were EUR 590.06 vs EUR 734.38 per person-year for vaccinated and unvaccinated cohorts, respectively. This results in a difference of EUR 144.32 per person-year and a total cost reduction of EUR 1,034,474.00 in the vaccinated cohort during the same follow-up period.

## Discussion

The ROUTINE-COV19 study evaluates the potential benefit of seasonal COVID-19 vaccination on clinical outcomes and healthcare utilisation in Germany using real-world data from SHI claims in two German states. Based on a matched cohort of over 146,000 individuals, the analysis provides robust evidence on the effectiveness of seasonal COVID-19 vaccination within routine care, following its formal integration into standard practice in April 2023. The study demonstrates that vaccination was associated with substantial reductions in adverse clinical outcomes and healthcare costs during the 2023 autumn vaccination season, reflecting a period of relatively high SARS-CoV-2 circulation in the endemic phase.

According to STIKO’s recommendations [5,20], the primary goal of COVID-19 vaccination is the prevention of severe disease progression, hospitalisations, deaths and long-term consequences of COVID-19 across the entire population. The demographic and clinical profile of the vaccinated cohort, characterised by higher age and a higher prevalence of comorbidities, was consistent with STIKO’s recommendation [5] for annual seasonal COVID-19 vaccinations of older adults and individuals at increased risk of severe COVID-19. The RKI estimates that 5–10% of individuals infected with SARS-CoV-2 develop long COVID and therefore experience persistent health issues [21-23]. In our study, vaccinated individuals consistently showed lower rates of long COVID diagnoses and respiratory infections, with RR of 0.43 and 0.91, respectively. They also experienced fewer COVID-19-, respiratory- and cardiovascular-related hospitalisations. Notably, vaccination was associated with ca 25% lower all-cause mortality (HR: 0.76), a finding consistent across all sensitivity analyses. However, this association should be interpreted with caution, as residual confounding and healthy vaccinee bias may have contributed to the observed difference. Mortality during COVID-19-related hospitalisations also appeared lower in the vaccinated cohort. However, this finding should be interpreted cautiously, as estimates were based on a limited number of events, with wider confidence intervals and less consistent results across sensitivity analyses. 

Similar biases have been described in studies on influenza vaccination, where vaccinated individuals were found to engage more frequently in preventive care and early healthcare-seeking behaviour [24,25]. The higher outpatient care utilisation observed among vaccinated individuals in this study supports this interpretation. Similar behavioural confounding may also affect broader, non-specific outcomes such as CVD-related or hospitalisations due to respiratory infections.

Our findings underscore the protective role of vaccination not only against acute SARS-CoV-2 infection but also against more severe outcomes, including hospitalisation and death. They align with the prospective German COViK hospital-based case-control vaccine effectiveness study, which reported 55–96% effectiveness, depending on the number of doses of COVID-19 vaccines in preventing hospitalisation caused by COVID-19 during Omicron variant circulation in 2021/22 [26]. Our results show a similar effect in the routine care context in Germany, underlining the continued relevance of seasonal vaccination in an endemic setting. These findings are also consistent with surveillance data reported to the ECDC, which show that higher COVID-19 vaccination uptake in older and high-risk populations is associated with reduced rates of severe outcomes, including hospitalisation and death, across European countries [27]. Furthermore, it complements data from cross-European reviews, which outline an association between broad vaccination and lower mortality in a post-pandemic context [28]. This study, as part of the broader European picture, further emphasises the importance of continued efforts in promoting vaccination among vulnerable groups.

The lower hospitalisation rates observed in this study also translate into substantially reduced inpatient care days and costs. For COVID-19-related hospitalisations alone, the cumulative inpatient cost savings across the 4-month observation period surpassed EUR 1 million in the AOK PLUS population. These results reflect findings from previous studies conducted outside of Germany, which highlight the value of vaccination in reducing hospitalisation risk or death and alleviating healthcare resource strain [29-32]. Our analysis also re-emphasises the epidemiological findings of the ROUTINE-COV19 study that people with cardiovascular risk profiles have a disproportionate risk of more severe outcomes from COVID-19 [14]. These findings are consistent with international studies showing that individuals with cardiovascular risk factors have an increased risk of severe COVID-19 outcomes, including hospitalisation and death [33], highlighting the importance of protecting these patients through vaccination. In response, the European Cardiology Society has also recently published new guidance on the particular value of protecting CVD patients against respiratory infections through vaccination [34].

Beyond direct health benefits, vaccinated working-age individuals incurred fewer COVID-19- and respiratory-related sick leave days, resulting in an estimated EUR 1.3 million reduction in indirect costs due to absence from work during the 4-month observation period of this study. The relevance of these findings is pertinent, as sick leave days resulting from respiratory infections represented the most common reason for absence from work in Germany in 2024 [35]. This highlights the broader economic relevance of vaccination from both healthcare payer and societal perspectives.

However, when considering the overall economic impact, vaccine administration costs should also be considered. During the inclusion period, a fixed reimbursement (‘Pauschale’) of EUR 15 per vaccine administration was in place. For the vaccinated individuals included in the matched cohort (base case), this corresponds to an estimated administration cost of approximately EUR 1.1 million, which is almost equivalent to the cost differences observed given fewer COVID-19–related hospitalisations. Vaccine acquisition cost could not be considered because of central procurement modalities of the vaccine in the analysed timeframe.

Within this study, we have evaluated various sensitivity scenarios. The observed consistency across these sensitivity analyses supports the validity of our results. As mentioned in systematic reviews, real-world studies are essential for assessing vaccination effectiveness, especially when randomised trials no longer reflect local epidemiological conditions [36]. However, several limitations must be considered. Firstly, despite robust matching and sensitivity analyses, residual confounding cannot be entirely excluded in this observational design, and therefore our findings should be interpreted in light of the methodology used. In this context, it is worth noting that vaccine uptake was relatively low during our study period, resulting in considerable differences in the number of vaccinated vs unvaccinated individuals available for matching. Secondly, the study population consisted of individuals ensured with AOK in the federal states of Saxony and Thuringia, together representing ca 4% of the German population. The age and sex distribution of the AOK-ensured population in these states is comparable to that of the national SHI population. There are no indications that the healthcare system or access to healthcare differ substantially between federal states. However, a regional bias cannot be completely ruled out. Furthermore, representativeness for Germany as a whole is limited to populations ensured under the SHI system and may not fully reflect privately ensured, potentially younger and healthier segments of the population. Thirdly, some outcomes may have been undercoded or misclassified in claims data. Moreover, cost and resource use through effort and losses associated with isolating patients with COVID-19 in single- or double-rooms were not considered in this study. This may have a substantial additional impact on hospital workflows and costs for both hospitals and insurers. Fourthly, vaccination status was defined at inclusion and remained fixed throughout follow-up. A small proportion of individuals classified as unvaccinated (n = 2,417; 3.3%) received a COVID-19 vaccination during follow-up, which may have led to a slight underestimation of the observed associations. This potential bias was explored in Sensitivity Scenario C, which excluded these individuals, and results remained consistent with the base case. Fifthly, the follow-up period was limited to 4 months, which, while appropriate for assessing seasonal vaccine impact post-implementation, restricts long-term evaluation of effectiveness, durability, and outcomes with delayed onset. Consequently, the study can only provide a preliminary descriptive indication of long-term or post-COVID occurrence, as longer-term sequelae may not yet have been captured. The low incidence rates for this outcome should therefore be interpreted with caution as also indicated by the wide confidence intervals. Finally, sensitivity Scenarios C and D conditioned on future vaccination status, which may introduce selection bias [37]. Censoring individuals at the time of later vaccination would be a more appropriate approach for causal analyses [38]; however, given the descriptive nature of this study, these scenarios were considered exploratory.

## Conclusion

COVID-19 vaccination under routine care conditions in Saxony and Thuringia was associated with reductions in morbidity, mortality, healthcare resource use and indirect costs caused by sick leave. While acknowledging limitations inherent to the observational study design, these findings highlight the clinical and economic benefits of seasonal COVID-19 vaccination, particularly for individuals at increased risk of severe disease and for working-age populations with high social contact. Despite these benefits, vaccination coverage in key risk groups remains low. The results underscore the importance of improving uptake and support continued implementation of national vaccination recommendations.

## Data Availability

The datasets presented in this article are not readily available because of ethical and privacy restrictions. Requests to access the datasets should be directed to the corresponding author.

## Supplementary Data

884000 Supplement
