# Supplementary material for: Clinical and economic benefits of seasonal COVID-19 vaccination in Germany: results from the ROUTINE-COV19 Study, September 2022 to March 2024
Source: Euro Surveill. 2026 Apr 16;31(15):2500672. doi: 10.2807/1560-7917.ES.2026.31.15.2500672 (PMC13090756; doi:10.2807/1560-7917.ES.2026.31.15.2500672)
Supplement: Supplement [file 25-00672_MUELLER_Supplement.pdf]

This supplementary material is hosted by Eurosurveillance as supporting information alongside the article "Clinical and economic benefits of seasonal COVID-19 vaccination in Germany: results from the ROUTINE-COV19 Study, September 2022 to March 2024" on behalf of the authors who remain responsible for the accuracy and appropriateness of the content. The same standards for ethics, copyright, attributions and permissions as for the article apply. Eurosurveillance is not responsible for the maintenance of any links or email addresses provided therein

Supplemental Table 1: Overview of sensitivity scenarios

		Sensitivity A	Sensitivity B	Sensitivity C	Sensitivity D	Base Case - 1:3 matching	Sensitivity C - 1:3 matching
EBM & ICD-10 vaccination codes were considered		x					
Exclusion of COVID cases in the washout period (for unvaccinated, also in the 3-month inclusion period)			x				
Exclusion of cases vaccinated in the follow-up				x	x		x
Inclusion period (identification of vaccinated cases) restricted to October 2023					x		
Matching using nearest-neighbor matching based on estimated PS (without replacement and with a specified caliper of 0.0001)	1 to 1	x	x	x	x		
	1 to 3					x	x

This table summarizes the variations in inclusion and exclusion criteria applied across different sensitivity scenarios (A–D) and additional sensitivity analyses using 1:3 matching. "x" indicates the application of the respective criterion or method. (EBM – Einheitlicher Bewertungsmaßstab; unified coding system in ambulatory care; PS – Propensity Score)

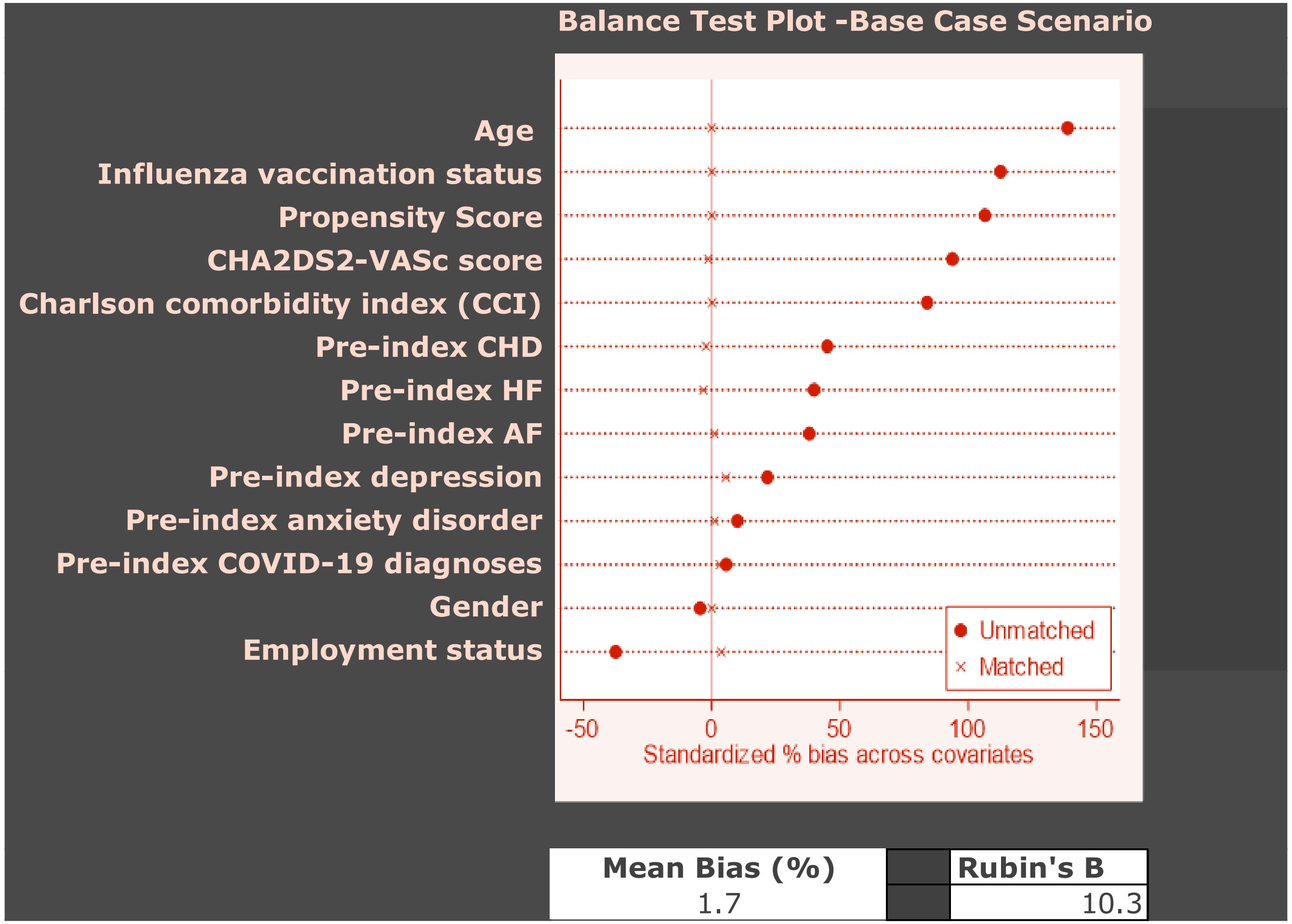

Supplemental Figure 1: Balance test plot for the base case scenario

Supplemental Table 2: Patient characteristics before and after matching in the defined sensitivity scenarios (AF- Atrial Fibrillation; HF – Heart Failure; CHD- Chronic Heart Disease)

		Sensitivity A				Sensitivity B				Sensitivity C				Sensitivity D				Base Case - 1:3 matching				Sensitivity C - 1:3 matching			
		Unmatched		Matched		Unmatched		Matched		Unmatched		Matched		Unmatched		Matched		Unmatched		Matched		Unmatched		Matched	
		Vac.	Unvac.	Vac.	Unvac.	Vac.	Unvac.	Vac.	Unvac.	Vac.	Unvac.	Vac.	Unvac.	Vac.	Unvac.	Vac.	Unvac.	Vac.	Unvac.	Vac.	Unvac.	Vac.	Unvac.	Vac.	Unvac.
	**Patients - N:**	72,686	3,140,006	72,685	72,685	71,862	3,066,003	71,861	71,861	73,037	3,136,060	73,035	73,035	54,781	3,136,060	54,775	54,775	73,037	3,136,060	73,003	219,009	73,067	3,145,952	73,050	219,150
**Variable**																									
Age	Mean	71.86	44.39	71.86	71.87	71.93	44.34	71.93	71.94	71.86	44.34	71.86	71.87	72.63	44.34	3.15	3.15	71.86	44.34	71.85	71.87	71.85	44.43	71.85	71.87
	SD	13.56	24.46	13.56	13.55	13.48	24.56	13.48	13.47	13.54	24.45	13.54	13.53	13.22	24.45	2.78	2.78	13.54	24.45	13.54	13.51	13.55	24.47	13.55	13.54
	Median	73.00	45.00	73.00	73.00	73.00	45.00	73.00	73.00	73.00	45.00	73.00	73.00	73.00	45.00	3.00	3.00	73.00	45.00	73.00	73.00	73.00	45.00	73.00	73.00
Female Sex	n	36,467	1,646,076	36,467	36,474	36,015	1,601,737	36,015	36,026	36,634	1,644,661	36,634	36,642	27,599	1,644,661	35,804	35,789	36,634	1,644,661	36,634	109,938	36,647	1,649,594	36,647	110,018
	Percent	50.2%	52.4%	50.2%	50.2%	50.1%	52.2%	50.1%	50.1%	50.2%	52.4%	50.2%	50.2%	50.4%	52.4%	65.4%	65.3%	50.2%	52.4%	50.2%	50.2%	50.2%	52.4%	50.2%	50.2%
Employment status																									
Employee/self-payer	n	12,847	1,489,531	12,847	14,005	12,479	1,435,753	12,479	13,611	12,892	1,489,431	12,892	14,086	8,775	1,489,431	16	18	12,892	1,489,431	12,892	42,290	12,898	1,491,441	12,898	42,280
	Percent	17.7%	47.4%	17.7%	19.3%	17.4%	46.8%	17.4%	18.9%	17.7%	47.5%	17.7%	19.3%	16.0%	47.5%	0.0%	0.0%	17.7%	47.5%	17.7%	19.3%	17.7%	47.4%	17.7%	19.3%
Pensioner/retiree	n	56,884	798,564	56,883	55,929	56,447	786,421	56,446	55,466	57,172	794,726	57,170	56,059	43,893	794,726	80	78	57,172	794,726	57,138	168,314	57,191	802,076	57,174	168,478
	Percent	78.3%	25.4%	78.3%	76.9%	78.5%	25.6%	78.5%	77.2%	78.3%	25.3%	78.3%	76.8%	80.1%	25.3%	0.1%	0.1%	78.3%	25.3%	78.3%	76.9%	78.3%	25.5%	78.3%	76.9%
Unemployed	n	2,010	183,928	2,010	1,876	1,997	181,596	1,997	1,908	2,028	183,926	2,028	1,927	1,480	183,926	3	2	2,028	183,926	2,028	5,737	2,030	184,268	2,030	5,695
	Percent	2.8%	5.9%	2.8%	2.6%	2.8%	5.9%	2.8%	2.7%	2.8%	5.9%	2.8%	2.6%	2.7%	5.9%	0.0%	0.0%	2.8%	5.9%	2.8%	2.6%	2.8%	5.9%	2.8%	2.6%
Rehabilitator	n	48	3,931	48	61	47	3,778	47	48	48	3,929	48	55	27	3,929	0	0	48	3,929	48	171	48	3,941	48	158
	Percent	0.1%	0.1%	0.1%	0.1%	0.1%	0.1%	0.1%	0.1%	0.1%	0.1%	0.1%	0.1%	0.0%	0.1%	0.0%	0.0%	0.1%	0.1%	0.1%	0.1%	0.1%	0.1%	0.1%	0.1%
Insured family member	n	897	664,052	897	814	892	658,455	892	828	897	664,048	897	908	606	664,048	1	1	897	664,048	897	2,497	900	664,226	900	2,539
	Percent	1.2%	21.1%	1.2%	1.1%	1.2%	21.5%	1.2%	1.2%	1.2%	21.2%	1.2%	1.2%	1.1%	21.2%	0.0%	0.0%	1.2%	21.2%	1.2%	1.1%	1.2%	21.1%	1.2%	1.2%
Charlson comorbidity index	Mean	2.93	0.97	2.93	2.93	2.93	0.96	2.93	2.93	2.94	0.96	2.94	2.94	3.15	0.96	8.05	7.97	2.94	0.96	2.94	2.93	2.94	0.97	2.94	2.93
	SD	2.73	1.88	2.73	2.72	2.72	1.87	2.72	2.72	2.73	1.88	2.73	2.73	2.78	1.88	9.32	9.22	2.73	1.88	2.72	2.72	2.73	1.88	2.72	2.71
	Median	2.00	0.00	2.00	2.00	2.00	0.00	2.00	2.00	2.00	0.00	2.00	2.00	3.00	0.00	6.00	6.00	2.00	0.00	2.00	2.00	2.00	0.00	2.00	2.00
Elixhauser comorbidity index	Mean	7.44	2.28	7.44	7.42	7.42	2.27	7.42	7.43	7.45	2.28	7.45	7.42	8.06	2.28	1.80	1.79	7.45	2.28	7.45	7.44	7.45	2.29	7.45	7.40
	SD	9.08	6.15	9.08	9.03	9.06	6.12	9.06	9.05	9.09	6.15	9.09	9.06	9.33	6.15	1.26	1.28	9.09	6.15	9.08	9.05	9.09	6.17	9.08	9.03
	Median	5.00	0.00	5.00	5.00	5.00	0.00	5.00	5.00	5.00	0.00	5.00	5.00	6.00	0.00	2.00	2.00	5.00	0.00	5.00	5.00	5.00	0.00	5.00	5.00
CHA_2DS_2-VASc score	Mean	1.70	0.62	1.70	1.70	1.70	0.62	1.70	1.70	1.70	0.62	1.70	1.70	1.80	0.62	0.71	0.71	1.70	0.62	1.70	1.70	1.70	0.63	1.70	1.70
	SD	1.25	1.02	1.25	1.25	1.25	1.02	1.25	1.25	1.25	1.02	1.25	1.25	1.26	1.02	0.61	0.61	1.25	1.02	1.25	1.25	1.25	1.02	1.25	1.25
	Median	2.00	0.00	2.00	2.00	2.00	0.00	2.00	2.00	2.00	0.00	2.00	2.00	2.00	0.00	1.00	1.00	2.00	0.00	2.00	2.00	2.00	0.00	2.00	2.00
Influenza vaccination	n	46,457	496,780	46,456	46,443	45,944	482,171	45,943	45,925	46,666	493,138	46,664	46,643	35,810	493,138	9,072	8,647	46,666	493,138	46,632	139,787	46,678	498,989	46,661	140,034
	Percent	63.9%	15.8%	63.9%	63.9%	63.9%	15.7%	63.9%	63.9%	63.9%	15.7%	63.9%	63.9%	65.4%	15.7%	16.6%	15.8%	63.9%	15.7%	63.9%	63.8%	63.9%	15.9%	63.9%	63.9%
Pre-index AF	n	10,959	127,761	10,959	10,778	10,809	124,079	10,809	10,640	11,014	127,326	11,013	10,902	9,074	127,326	10,842	11,196	11,014	127,326	10,995	32,568	11,017	128,512	11,008	32,313
	Percent	15.1%	4.1%	15.1%	14.8%	15.0%	4.0%	15.0%	14.8%	15.1%	4.1%	15.1%	14.9%	16.6%	4.1%	19.8%	20.4%	15.1%	4.1%	15.1%	14.9%	15.1%	4.1%	15.1%	14.7%
Pre-index HF	n	12,941	166,047	12,940	13,748	12,751	161,691	12,750	13,479	13,046	166,093	13,044	13,743	10,846	166,093	12,997	13,389	13,046	166,093	13,023	41,412	13,048	167,497	13,035	41,328
	Percent	17.8%	5.3%	17.8%	18.9%	17.7%	5.3%	17.7%	18.8%	17.9%	5.3%	17.9%	18.8%	19.8%	5.3%	23.7%	24.4%	17.9%	5.3%	17.8%	18.9%	17.9%	5.3%	17.8%	18.9%
Pre-index CHD	n	15,882	204,649	15,881	16,730	15,717	199,913	15,716	16,333	16,008	204,335	16,006	16,694	13,003	204,335	9,505	8,382	16,008	204,335	15,985	49,862	16,011	206,239	15,999	49,848
	Percent	21.9%	6.5%	21.8%	23.0%	21.9%	6.5%	21.9%	22.7%	21.9%	6.5%	21.9%	22.9%	23.7%	6.5%	17.4%	15.3%	21.9%	6.5%	21.9%	22.8%	21.9%	6.6%	21.9%	22.7%
Pre-index depression	n	11,933	288,970	11,933	10,785	11,750	279,175	11,750	10,676	12,011	288,806	12,011	10,824	9,507	288,806	5,169	4,887	12,011	288,806	12,005	32,268	12,019	290,284	12,013	32,694
	Percent	16.4%	9.2%	16.4%	14.8%	16.4%	9.1%	16.4%	14.9%	16.4%	9.2%	16.4%	14.8%	17.4%	9.2%	9.4%	8.9%	16.4%	9.2%	16.4%	14.7%	16.4%	9.2%	16.4%	14.9%
Pre-index anxiety disorder	n	6,544	198,999	6,544	6,334	6,451	192,166	6,451	6,258	6,586	199,065	6,586	6,413	5,169	199,065	0	0	6,586	199,065	6,583	19,080	6,588	199,897	6,586	19,063
	Percent	9.0%	6.3%	9.0%	8.7%	9.0%	6.3%	9.0%	8.7%	9.0%	6.3%	9.0%	8.8%	9.4%	6.3%	0.0%	0.0%	9.0%	6.3%	9.0%	8.7%	9.0%	6.4%	9.0%	8.7%

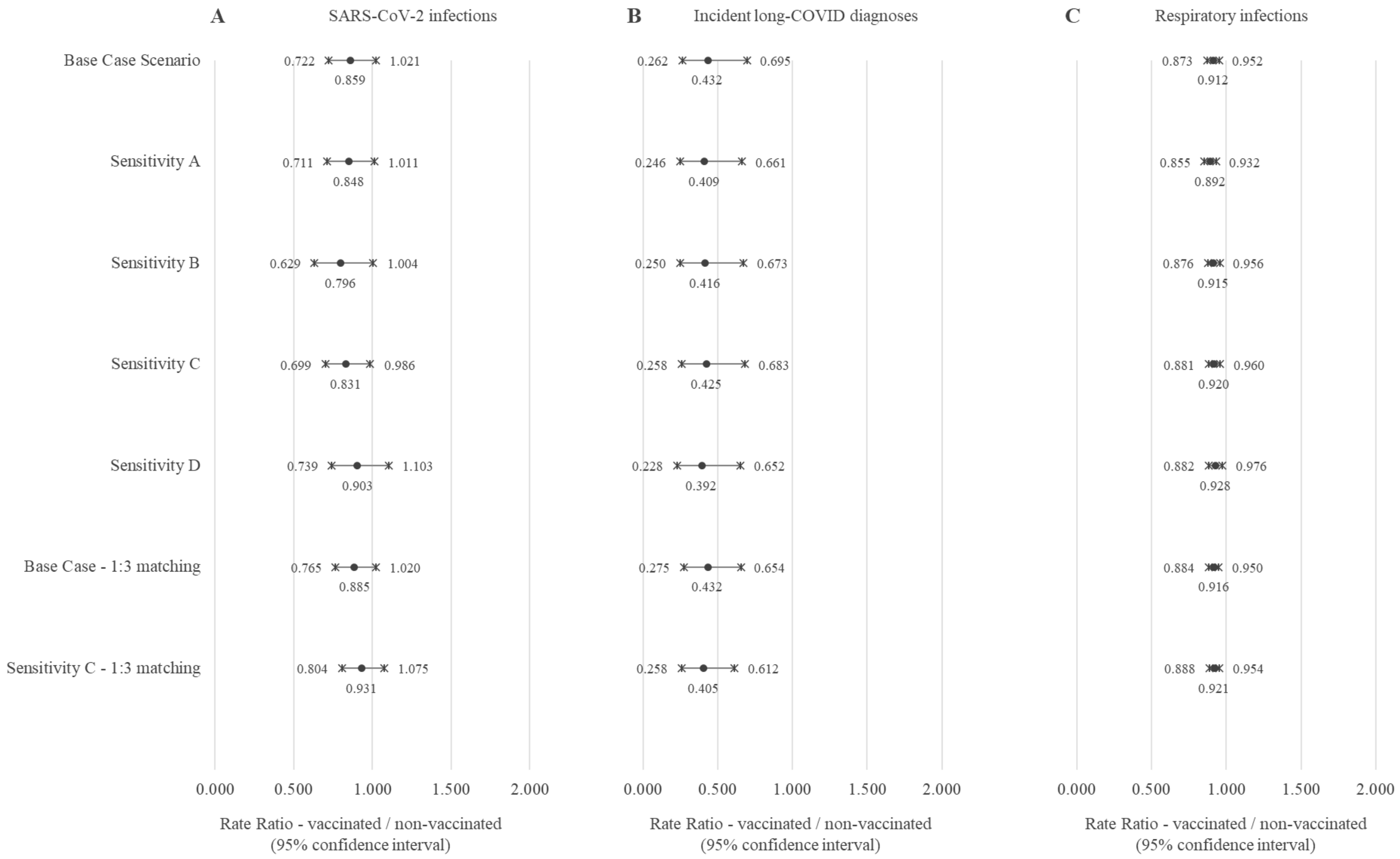

Abbreviations: COVID-19 – Coronavirus Disease 2019, Long COVID – Post-COVID-19 condition as defined by ICD-10-GM U09.9!, SARS-CoV-2 – Severe Acute Respiratory Syndrome Coronavirus 2

Supplemental Figure 2: Forest plot showing rate ratios based on observed events per person-time during the follow-up period for different sensitivity scenarios, comparing the vaccinated and non-vaccinated cohorts for (A) SARS-CoV-2 infections, (B) incident long COVID diagnoses, and (C) respiratory infections

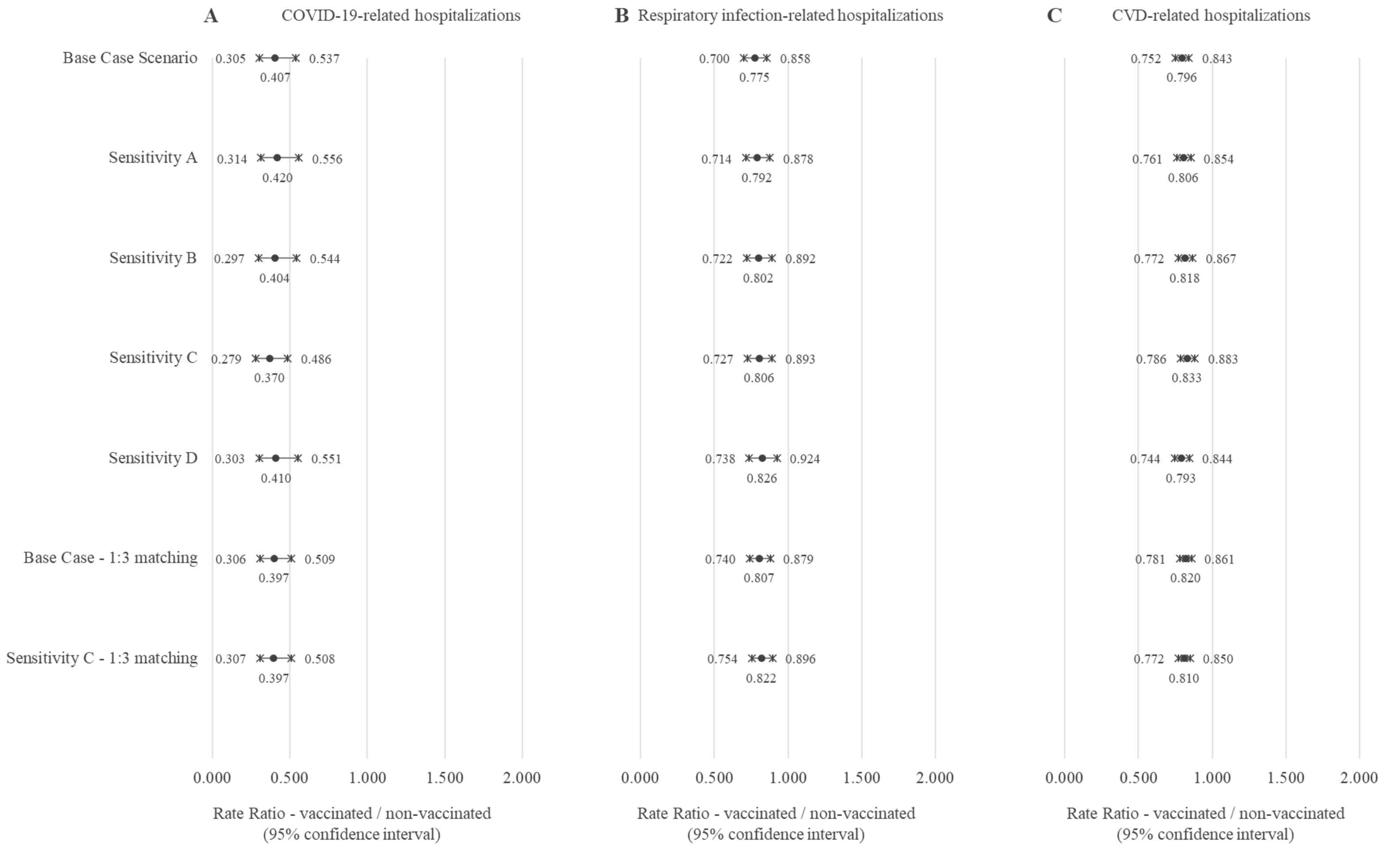

Abbreviations: COVID-19 – Coronavirus Disease 2019, CVD – Cardiovascular Disease

Supplemental Figure 3: Forest plot showing rate ratios based on observed events per person-time during the follow-up period for different sensitivity scenarios, comparing the vaccinated and non-vaccinated cohorts for (A) COVID-19-related hospitalizations, (B) respiratory infection-related hospitalizations, and (C) cardiovascular disease-related hospitalizations

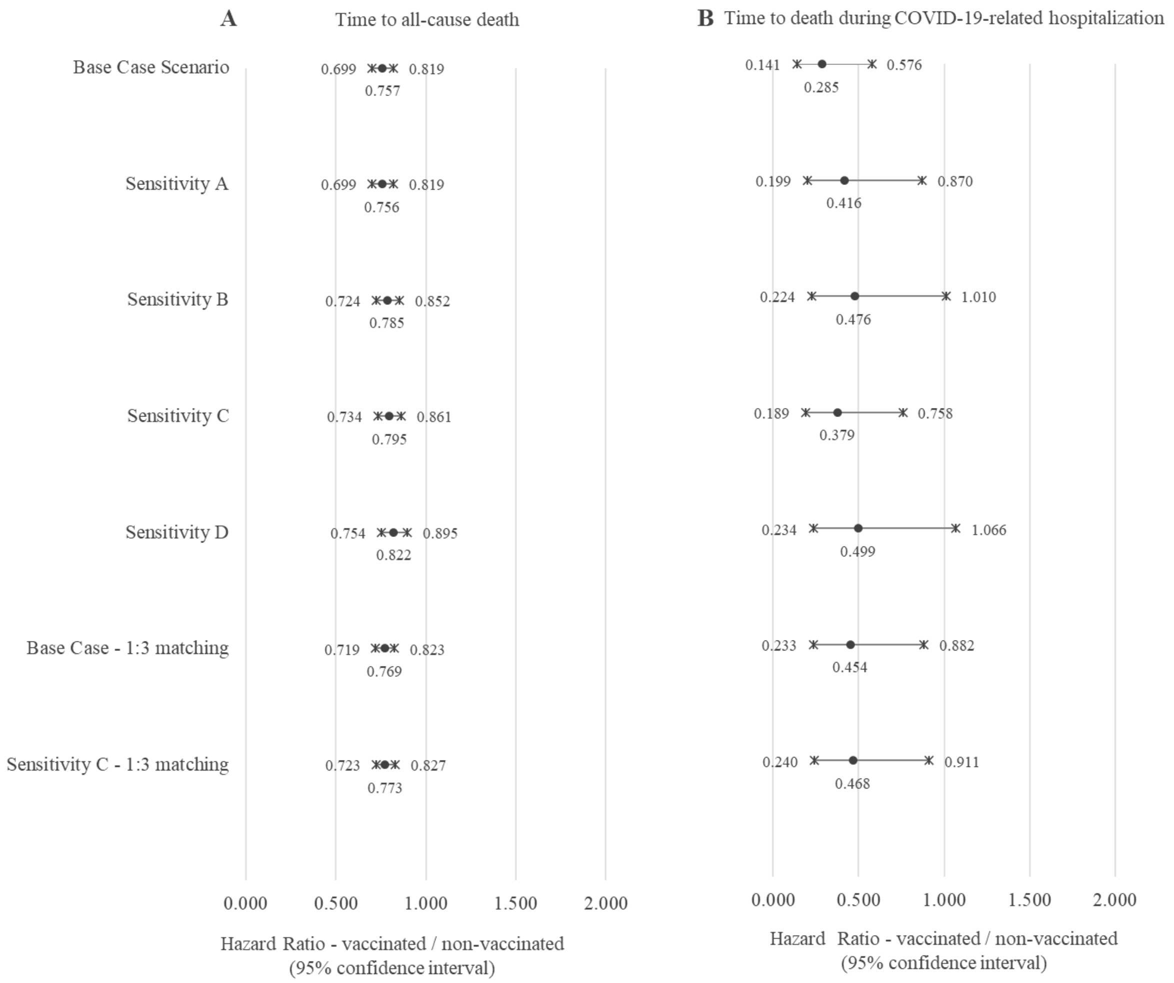

Abbreviations: COVID-19 – Coronavirus Disease 2019

Supplemental Figure 4: Forest plot showing hazard ratios for time to death comparing the vaccinated and non-vaccinated cohorts in the different sensitivity scenarios: (A) All-cause death, (B) death during COVID-19-related hospitalizations
